# Knowledge, attitudes and influencers of cat owners in North America around antimicrobials and antimicrobial stewardship

**DOI:** 10.1177/1098612X221090456

**Published:** 2022-04-26

**Authors:** Madeleine R Stein, J Scott Weese, Jason W Stull, J Trenton McClure, Michelle Evason

**Affiliations:** 1 Department of Companion Animals, Atlantic Veterinary College, University of Prince Edward Island, Prince Edward Island, Canada; 2 Department of Clinical Sciences, Cummings School of Veterinary Medicine, Tufts University, MA, USA; 3 Department of Health Management, Atlantic Veterinary College, University of Prince Edward Island, Prince Edward Island, Canada; 4 Department of Pathobiology, Ontario Veterinary College, University of Guelph, Guelph, ON, Canada; 5 IDEXX Laboratories, Portland, ME, USA

**Keywords:** Antimicrobials, survey, resistance, conjoint analysis

## Abstract

**Objectives:**

The primary aims of this study were to determine preferences of North American cat owners when they are prescribed an antimicrobial for their cat with regard to cost, method of administration and the importance of antibiotics for treating infections in people, and to establish baseline knowledge, attitudes and influencers of cat owners on antimicrobial resistance and stewardship.

**Methods:**

An online questionnaire was used for data collection from two cat-owner groups: US cat owners and Canadian cat owners. Participants were queried on antimicrobial resistance and stewardship, and their preferences for their own cat when prescribed an antimicrobial, with respect to cost, method of drug administration and the importance of a drug for treating infections in people. Responses were evaluated through conjoint analysis and Likert-type questions. Data were analyzed using descriptive and analytic statistics.

**Results:**

A total of 630 complete responses were included in the final analysis. Cost (37%) and method of administration (38%) were of similar participant preference when assessed using conjoint analysis. The importance of a drug for treating infections in people was lower priority (21%). The majority of cat owners preferred an antimicrobial that was ‘very important’ in treating human infections. A low proportion (21%) of participants responded that antimicrobial use in pets posed a risk to humans. Participants with a university education were more likely to respond that antimicrobial use in pets was a concern for people (31%; *P* <0.001).

**Conclusions and relevance:**

Cat owners prioritize antimicrobial cost and method of administration equally. Few cat owners recognized the human antimicrobial resistance risks associated with antimicrobial use in pets.

## Introduction

Transfer of antimicrobial-resistant pathogens between pets and their owners is increasingly recognized worldwide.^1^ In addition, there is concern for the negative impacts of resistant infections on animal welfare.^2^ Antimicrobial stewardship (AMS) has gained traction in all medical fields, including veterinary medicine. There has been a shift from AMS that focuses on antimicrobial prescribers to a more holistic approach, including the involvement and education of human patients and pet owners.^3^

Pet owner cost constraint is frequently perceived by veterinarians to be a significant barrier to AMS efforts.^4,5^ In feline practice, surveys suggest cats are less likely than dogs to be taken to the veterinarian and also have less money spent on their care than dogs.^6,7^ This may indicate that cost concerns may be more of a barrier for cat owners than for dog owners. However, previous surveys of pet owners have provided conflicting evidence on willingness to spend money on veterinary care.^8–10^

Compliance with treatment is a concern in both human and veterinary medicine.^11^ One study on dog owners indicated a preference for not administering oral medications.^8^ Compared with dogs, cats are perceived to be more difficult to administer oral medications to, and are frequently prescribed injectable medications to alleviate this challenge.^12–16^ Surveys specific to cat owners are required to determine the extent that method of administration influences owner preferences on antimicrobial prescription for their cat.

The few studies that have been performed in companion and livestock owners have demonstrated that awareness and understanding of antimicrobial resistance (AMR) in animals is limited and often of low priority.^8,10,17,18^ Similarly, it is recognized that the general public has a superficial understanding of AMR in human medicine, and, due to this, AMS programs that include patient education are more likely to be successful.^19^ It follows that education of pet owners in companion animal medicine should be included in veterinary AMS programs. As such, baseline levels of pet owner AMR and AMS knowledge need to be established to assist with program development.

A previous study produced by our research team in North American dog owners explored knowledge, attitudes and influencers (KAIs) of antimicrobial drug attributes using choice-based conjoint analysis and multiple-choice questions.^8^ This cat owner work aims to complement our canine study, using the same techniques in a similar subset of cat owners. The primary objectives of this study were to quantify the influence of cost, ease of administration and drug importance in human medicine on cat owner selection of antimicrobials; and explore associations between demographics (eg, respondent age and sex) on owner understanding of AMR in human and veterinary medicine, and their perception of its importance. We hypothesized that cat owners would identify cost and method of administration as the most important attributes in a drug; and that cat owners would have a limited knowledge of AMR and AMS in a veterinary setting.

## Materials and methods

### Data collection

Two participant groups (US and Canadian cat owners) were recruited using an online survey platform (Qualtrics). This was a prospective study using an online questionnaire targeting Canadian and US cat owners. Survey data were initially collected in September 2019, but, due to an error during initial collection, Canadian data were recollected using a different study population in July 2020. In addition to conjoint style questions, owners were asked a series of multiple-choice questions consisting of basic demographic information, KAIs surrounding antimicrobial prescription in their own cat and two questions about AMR in human and veterinary medicine.

Conjoint analysis – a novel survey technique adapted from marketing for use in clinical research – was used to determine pet owner preferences with regard to drug attributes (features). In marketing, participants are provided with a series of choices between two or more products with varying features (eg, cost or color) to assess their preference for these features. In our study, participants were provided with a series of choices between antimicrobials for their pet. These choices consisted of a hypothetical scenario in which participants were asked to pick which of two equally effective antimicrobials they would prefer if their cat had a urinary tract infection. The scenario was used to assess three antimicrobial features (eg, cost), each of which had between three and four levels (eg, $25, $45 or $80). A summary of the features and potential levels are presented in Table 1. These were presented in a series of 10 choices between two randomly generated antimicrobials to each participant, with participants selecting their preferred option. Through these 10 questions, participants provided information about which features of an antimicrobial drug were important to them.Bayesian hierarchical analysis was used to calculate a numerical score that allowed quantification of participant preference. A positive or negative numerical score (utility value) was calculated for the levels of each feature, with a positive association between the utility value and a participant’s likelihood to pick a drug with the level being assessed. The difference between the highest (most desired) and lowest (least desired) levels of a feature for an individual participant is called the preference score, and indicates the importance of that feature as it indicates the impact it has on the participant’s choice. A larger difference between the highest and lowest levels, and so a higher preference score, indicates this feature has a larger impact on decision-making. The preference share for each feature is the mean preference score of that feature divided by the sum of the mean preference scores for all three features assessed; this indicates how much each feature affects overall decision-making vs the other two features.

**Table 1:** Summary of the features and levels of the conjoint section in a survey to assess knowledge, attitudes and influencers of cat owners in North America around antimicrobials and antimicrobial stewardship

Feature	Level
Cost	$25 USD/CAD
	$45 USD/$50 CAD
	$80 USD/$90 CAD
How the antimicrobial is given	Injected once by your veterinarian
	Oral (pill or liquid) once a day for 5 days
	Oral (pill or liquid) twice a day for 5 days
	Oral (pill or liquid) three times a day for 5 days
Importance of the drug for treating infections in people	Very important
	Somewhat important
	Not important

USD = US dollars; CAD = Canadian dollars

### Study population

The two participant groups consisted of convenience samples, recruited via a pool of survey-takers provided by the survey platform Qualtrics. Participants were offered a small compensation for completing the survey in the form of points towards a rewards scheme. Inclusion criteria were that participants had to be a minimum of 18 years of age, reside in either the USA or Canada, and have owned or looked after a cat within the previous year. A sample size of a minimum of 300 participants per group was selected to facilitate comparison between Canadian and US cat owners. This was twice the recommended number for a conjoint project with this number of levels and features.

### Statistical analysis

Commercially available software (Minitab Statistical Software) was used for all statistical analysis. All continuous variables (ie, conjoint data) were assessed for normality using Anderson–Darling normality tests. The majority of variables were found to be non-normally distributed; consequently, all continuous data were expressed as medians and upper and lower quartiles. The data for the feature ‘importance of a drug for treating people’ were found to be normally distributed, so parametric tests were performed on these data where appropriate.

Multiple-choice questions with five possible responses were recoded into two or three possible responses (of roughly equal size) to address small sample sizes. Age, household income and perceived importance of AMR in human medicine were retained in their original categories.

Pearson’s χ^2^ tests were used to identify associations between categorical variables. Investigation into the association between age and household income, and response to the question ‘How important do you think antibiotic resistance is in human medicine?’ was not possible owing to small cell sizes and computational power required. For these comparisons, age and household income were dichotomized to allow Pearson’s χ^2^ testing. A one-way ANOVA was performed on level of education and participant utility value for the level ‘not important’ of the feature ‘importance of a drug for treating people’.

A Bonferroni adjustment to correct for multiple comparisons and reduce the likelihood of type 1 errors was performed. Comparisons between the demographic makeup of the two participant groups were considered significant at *P* <0.0125, and differences between participant demographics and response to the questions ‘How important do you think antibiotic resistance is in human medicine?’ and ‘Do you think antibiotic use in pets poses a risk to humans?’ were considered significant at *P* <0.01. All other tests were considered statistically significant at *P* <0.05.

### Ethical approval

Ethical approval for human research was obtained from the Research Ethics Boards of the University of Prince Edward Island (#6008583) and the University of Guelph (#19-03-13).

## Results

A total of 630 surveys were completed (Canadian participants, n = 315; US participants, n = 315) and all were included in the final analysis.

### Demographic data

Demographic data of all participants are summarized in Table 2. The two groups significantly differed in age, with the Canadian group having a higher proportion of participants in the older age categories than the US group (*P* <0.001).

**Table 2:** Participant demographics from a survey to assess knowledge, attitudes and influencers of cat owners in North America around antimicrobials and antimicrobial stewardship summarised by total population and study group

	Canada†	USA†	Total†
Sex			
Male	125 (40)	97 (31)	222 (35)
Female	190 (60)	216 (69)	406 (65)
Total	315	313	628
			*P* = 0.023*
Age (years)			
18–25	10 (3.2)	39 (12)	49 (7.8)
26–35	57 (18)	69 (22)	126 (20)
36–50	85 (27)	108 (34)	193 (31)
51–65	111 (35)	73 (23)	184 (29)
>65	52 (17)	26 (8.3)	78 (12)
Total	315	315	630
			*P <*0.001
Approximate household income ($)			
<50,000	120 (39)	132 (42)	252 (40)
51,000–100,000	122 (39)	100 (32)	222 (35)
101,000–200,000	54 (17)	57 (18)	111 (18)
>200,000	9 (2.9)	8 (2.5)	17 (2.7)
Prefer not to answer	10 (3.2)	18 (5.7)	28 (4.4)
Total	315	315	630
			*P* = 0.270
Highest level of education			
High school	79 (25)	108 (34)	187 (30)
Community college	105 (33)	87 (28)	192 (30)
University degree	131 (42)	120 (38)	251 (40)
Total	315	315	630
			*P* = 0.036

Data are n (%) unless otherwise stated. *P* values demonstrate differences in demographic distributions between the participant groups, as calculated by Pearson’s χ^2^ tests

* *P* values indicate differences between the two participant groups

† Percentages in the columns may not add up to 100 due to rounding

### Choice-based conjoint analysis

Both the US and Canadian participant groups considered cost and method of administration to be of similar importance (Table 3). The median preference score for cost was 4.13, with $25 level with the highest median utility value (1.97) and $80 with the lowest (–2.19). The median preference score for method of administration was 3.86. The level with the highest median utility value was ‘injected once by your veterinarian’ (1.94) and the level with the lowest utility value (–1.78) was ‘oral (pill or liquid) three times a day for 5 days’. The percentage preference share for cost and method of administration were 37% and 38%, respectively.

**Table 3:** Summary of results of conjoint analysis in a survey to assess knowledge, attitudes and influencers of cat owners in North America around antimicrobials and antimicrobial stewardship

	Canada		USA		Total	
	Median preference score^†^ (IQR)	Median utility value^*^	Median preference score (IQR)^†^	Median utility value*	Median preference score (IQR)†	Median utility value*
Method of administration	3.85 (1.80–6.95)		3.87 (2.33–6.18)		3.86 (2.16–6.51)	
Injected once by your veterinarian		1.85		2.04		1.94
Oral (pill or liquid) once a day for 5 days		0.315		0.400		0.326
Oral (pill or liquid) twice a day for 5 days		–0.435		–0.554		–0.495
Oral (pill or liquid) three times a day for 5 days		–1.85		–1.69		–1.78
Cost	4.34 (2.22–6.26)		3.94 (2.00–6.41)		4.13 (2.08–6.33)	
$25 USD/CAD		2.05		1.89		1.97
$45 USD/$50 CAD		0.247		0.168		0.214
$80 USD/$90 CAD		–2.26		–2.06		–2.19
Importance in human medicine	2.81 (1.31–4.90)		2.40 (1.23–3.58)		2.58 (1.30–4.00)	
Very important		1.23		1.19		1.20
Somewhat important		0.173		–0.00476		0.0863
Not important		–1.40		–1.17		–1.24

Median utility value for all levels and the median preference score (with first and third quartiles) for the three features are reported by recruitment group and total study population

* Positive or negative numerical value indicating participant’s preference for each level of each feature after all 10 conjoint questions were completed

† Difference between highest and lowest level for each feature

IQR = interquartile range; USD = US dollars; CAD = Canadian dollars

The feature with the lowest median preference score was importance of the drug for treating infections in people (2.58), with an overall preference share of 25%. The level with the highest utility value was ‘very important’ (1.20) and the level with the lowest utility value was ‘not important’ (–1.24). Participant education and utility value for ‘not important’ were not statistically significant (*P* = 0.088).

### KAIs

Participants were asked to use a Likert scale to rate the importance of four features (‘number of times a day a pill must be given’, ‘cost’, ‘the importance of the drug for treating infections in people’ and ‘whether or not you need to give your cat a pill’) when their cat was prescribed an antimicrobial. Results are fully summarized in Table 4. The results were largely consistent with the conjoint analysis, as the greatest number of participants viewed cost (74%; 95% confidence interval [CI] 70–77) and number of times a day a pill is administered (73%; 95% CI 69–76) to be of high importance (‘very important’ or ‘important’). The importance of the drug for treating infections in people had the lowest proportion of participants (57%; 95% CI 53–61) who considered it to be of high importance. The results between the US and Canadian study groups were largely consistent across all features.

**Table 4:** Summary of the number and proportion of participants in a survey of knowledge, attitudes and influencers of cat owners in North America around antimicrobials and antimicrobial stewardship who indicated that one of four factors that are taken into consideration when their cat is given an antimicrobial is ‘very important’ or ‘important’ to them

	Canada	US	Total	
	No of participants*	No of participants*	No of participants*	95% CI
Number of times a day a pill must be given	238 (76)	222 (71)	460 (73)	69.4–76.4
Cost	241 (76)	224 (71)	465 (74)	70.2–77.2
The importance of the drug for treating infections in people	198 (63)	163 (52)	361 (57)	53.3–61.2
Whether or not you need to give your cat a pill	228 (72)	198 (63)	426 (68)	63.8–61.3

Data are n (%)

* Percentages in the columns may not add up to 100 due to rounding

CI = confidence interval

### Knowledge of AMR

The majority of participants (56%) indicated that they considered AMR in human medicine to be very important. There was no significant association between response to this question and any of the five demographic categories (age, sex, household income, level of education, participant group) studied. A full summary of participant responses to the KAI questions is reported in Table 5 and Tables 1 and 2 in the supplementary material.

**Table 5:** Summary of participant responses to the questions ‘How important do you think antibiotic resistance is in human medicine?’ and ‘Do you think antibiotic use in pets poses a risk to people?’ in a survey to assess knowledge, attitudes and influencers of cat owners in North America around antimicrobials and antimicrobial stewardship

	Canada*	USA*	Total*
Importance of antibiotic resistance in humans			
Very important	175 (56)	177 (56)	352 (56)
Important	93 (30)	79 (25)	172 (27)
Slightly important	25 (7.9)	42 (13)	67 (11)
Don’t know	17 (5.4)	12 (3.8)	29 (4.6)
Not important at all	5 (1.6)	5 (1.6)	10 (1.6)
Total	315	315	630
Risk of antibiotic use in pets to humans			
Yes	59 (19)	75 (24)	138 (21)
Might or might not	103 (33)	117 (37)	222 (35)
No	153 (49)	123 (39)	276 (44)
Total	315	315	630

Data are n (%)

* Percentages in the columns may not add up to 100 due to rounding

Less than a quarter (21%) of participants indicated that they thought antimicrobial use (AMU) in pets posed a risk to humans, whereas nearly half (44%) thought that there was no risk. Associations were found between participant sex (*P* <0.002), household income (*P* <0.001) and level of education (*P* <0.001), and whether a participant thought it was likely that AMU in pets posed a risk to humans (sex: *P* <0.002; income: *P* <0.001; education; *P* <0.001). No associations were found between participant age or recruitment group and response to this question. In general, participants who were male, reported a higher household income or with a higher level of education were more likely to indicate that they thought AMU in pets posed a risk to humans.

## Discussion

Our work with North American cat owners indicates that cost and ease of antimicrobial administration share equal importance in cat owner preference of antimicrobial prescription, and these factors outweigh antimicrobial importance in human medicine.

It is widely believed that cats are more difficult to administer oral medications to than dogs.^12^ A previous study performed on a group of dog owners found that, while method of administration was important, cost had the greatest influence on dog owner antimicrobial preferences.^8^ This is in contrast to the findings of this study, where method of administration was found to have equal importance with cost, and medications that are injected only once as the most desirable to cat owners. Several recent surveillance studies performed to assess anti-microbial prescription in companion animal practice have found that cats are much more likely than dogs to be prescribed an injectable antimicrobial, which further supports our findings that cat owners may be reluctant to administer oral medications, or that veterinarians may preferentially use or recommend injectable medications because of perceived owner compliance issues or preferences.^13,15,16,20,21^ More research is needed to establish to what extent cat owner preferences and veterinarians’ perceptions of owner preferences influence prescription of injectable antimicrobials over oral antimicrobials, and to determine whether it is veterinarians themselves who encourage the use of this delivery method.

A possible reluctance by cat owners to administer oral medications has the potential to significantly impact AMS. For example, one long-acting injectable anti-microbial in veterinary practice, cefovecin,^14^ is a third-generation cephalosporin considered by the World Health Organization to be a ‘highest priority critically important antimicrobial’ in human medicine.^22^ These antimicrobials should be reserved for patients where no alternative treatments exist. Inability to administer oral medications would be a justified use, but it is likely that cefovecin is frequently prescribed in veterinary medicine with no clear justification.^14,15^ In addition, owing to the long-acting nature of this medication, it is not possible to use a short treatment course, even when it is indicated, or switch to a different antimicrobial without significant drug overlap if there is no initial response to treatment. Our data indicate that injectable antimicrobials were preferred by a large proportion of our study participants. Improved communication with clients regarding techniques to successfully administer oral medication, and why oral medication may be more appropriate in their pet, is warranted.

In addition to a marked preference for a single, long-acting antimicrobial injection, our conjoint analysis data indicated that there was an aversion to three-times-daily dosing of oral medication vs once- or twice-daily dosing. In the KAIs section, a large proportion of participants also valued the number of times a day a pill must be given. While there are few antimicrobials that require three-times-daily dosing, this lower preference for oral medication administration highlights an additional challenge in feline medicine. This preference warrants consideration when cats are being prescribed an antimicrobial as it likely impacts owner compliance. Studies performed in dogs indicate that many dog owners are not compliant with either the number of doses given or the intervals between doses, and that compliance decreases the more doses a day that are prescribed.^23–25^ Similar findings have been noted in human medicine.^26,27^ Given that there is a perception that cats are more difficult to medicate than dogs, and a similar aversion to increased daily dosing was found for dogs,^8^ it is reasonable to assume that similar or reduced levels of compliance may occur with increased frequency of dosing in cats.

Recent studies have resulted in a shift away from worries about incomplete antimicrobial courses, as there are more data to support the effectiveness of short course treatments.^28^ However, there is still concern that reduced client compliance with a prescribed treatment regimen may increase risks of AMR through inappropriate uses of remaining drugs.^29^ While, to date, no studies have been performed to assess compliance with treatment regimens in cats, extrapolations can be made from the human and canine literature. Dog owner compliance has been shown to increase when clients perceive veterinarians spend sufficient time with them during a consultation, they have an understanding of the disease they are treating, or the dosing regimen is adapted to suit the owner’s lifestyle.^24,25,30^ Similar findings have been demonstrated in human medicine.^31,32^ Our study demonstrates that future AMS programs in feline practice need to include tactics to better communicate with cat owners in order to ensure that compliance with treatment regimens is optimized.

Similar to findings in dog owners,^8^ and other studies exploring the knowledge of pet owners,^9,10^ there was a low understanding of AMR in a veterinary setting among the participants of this study. This was particularly apparent with cat owner knowledge of AMR risk and transmission between people and animals. Just over a quarter of the total study participants stated that they considered AMU in pets as ‘definitely’ or ‘probably’ posing a risk to humans. A lack of concern for the human risk of companion animal AMU is combined with the results of the conjoint analysis where the majority of participants would prefer a drug that was ‘very important’ in human medicine. These drugs should be reserved for critical cases where no alternative treatment exists, and are frequently not available for use in veterinary medicine due to the potential negative impact of their use. These questions did not directly address a risk of AMR transmission, instead referring to a general risk to humans. It may be that participants did not make an association between AMU in pets and AMR in people and were considering other risks. However, a lack of understanding of AMR transmission risk, even between two people in the same household, has been demonstrated in human studies,^33^ and it is likely that cat owners in this study have a similar gap in their knowledge. These findings demonstrate that there is a need for increased education of pet owners surrounding the connections between human and animal AMR, and the potential zoonotic risks of antimicrobial resistant diseases.

Significant associations were found between several demographic characteristics and KAIs on AMU in pets. The most notable of these was level of education, with a higher proportion of participants with a university level education indicating that AMU in pets was a risk to people. This was also the case in our canine study.^8^ Further study is required on the role of education in pet owner KAIs, and to determine the optimal way to disseminate AMR information to pet owners to aid further companion animal AMS efforts.

The primary limitation of this study is that the conjoint scenarios are hypothetical and may not accurately reflect the decisions that a cat owner may make when facing the emotional stressors of a sick pet. The nature of the survey, which was designed to be quantitative as opposed to qualitative (eg, small group interviews) also makes it difficult to draw conclusions about the motivations behind participant answers, especially for the conjoint analysis. The sample size of this survey is comparatively large when other pet owner studies are considered, but it is still only a fraction of the total North American cat owning population. While efforts were made to gather a representative sample, caution must be exercised when extrapolating these data.

## Conclusions

As in our canine work, knowledge of AMR in veterinary medicine was limited in cat owners. Further research is needed on methods of communication to veterinary clients specific to AMS, and additional efforts towards establishment of baseline knowledge of AMR and AMS within the veterinary community. This study, combined with our canine project, provides practical considerations for companion animal veterinarians when prescribing antimicrobials to aid compliance, and is a One Health ‘call to action’ towards AMR and AMS education of pet owners.

## Supplemental Material

Table 1 Click here for additional data file. Participant responses to the question ‘How important do you think antibiotic resistance is in human medicine?’ from a survey to assess knowledge, attitudes and influencers of cat owners in North America around antimicrobials and antimicrobial stewardship summarized by demographic group.

Table 2 Click here for additional data file. Participant responses to the question ‘Do you think antibiotic use in pets poses a risk to people?’ from a survey to assess knowledge, attitudes and influencers of cat owners in North America around antimicrobials and antimicrobial stewardship summarized by demographic group.
