# Supplementary material for: Knowledge, attitudes and influencers of cat owners in North America around antimicrobials and antimicrobial stewardship
Source: J Feline Med Surg. 2022 Apr 26;24(6):e90–7. doi: 10.1177/1098612X221090456 (PMC9161435; doi:10.1177/1098612X221090456)
Supplement: Table 1 [file sj-docx-1-jfm-10.1177_1098612X221090456.docx]

**Supplementary Materials**

**Knowledge, attitudes and influencers of North American cat-owners around antimicrobials and antimicrobial stewardship**

Supplementary table 1. Participant response to the question ‘How important do you think antibiotic resistance is in human medicine?’ from a survey to assess knowledge, attitudes and influencers of cat owners in North America around antimicrobials and antimicrobial stewardship summarised by demographic group. The *p* values demonstrate differences in distributions of response between the demographic groups, as calculated by Pearson’s χ^2^ tests.

|  | **Very important** | **Important** | **Slightly important** | **Don’t know** | **Not at all important** | **Total** |
| --- | --- | --- | --- | --- | --- | --- |
|  | **Gender** | | | | | |
| Female | 218 (62%) | 107 (62%) | 50 (75%) | 22 (76%) | 9 (90%) | **406 (65%)** |
| Male | 132 (38%) | 65 (38%) | 17 (25%) | 7 (24%) | 1 (10%) | **222 (35%)** |
| **Total** | **350** | **172** | **67** | **29** | **10** | **628** |
|  |  |  |  |  |  | **p=0.071** |
|  | **Age** | | | | | |
| 18-25 years old | 27 (7.7%) | 5 (2.9%) | 11 (16%) | 4 (14%) | 2 (20%) | **49 (7.8%)** |
| 26-35 years old | 65 (18%) | 38 (22%) | 16 (24%) | 6 (21%) | 1 (10%) | **126 (20%)** |
| 36-50 years old | 106 (30%) | 55 (32%) | 19 (28%) | 8 (28%) | 5 (50%) | **193 (31%)** |
| 51-65 years old | 102 (29%) | 54 (31%) | 20 (30%) | 7 (24%) | 1 (10%) | **184 (29%)** |
| > 65 years old | 52 (15%) | 20 (12%) | 1 (1.5%) | 4 (14%) | 1 (10%) | **78 (12%)** |
| **Total** | **352** | **172** | **67** | **29** | **10** | **630** |
|  |  |  |  |  |  | **p=0.134**^§^ |
|  | **Approximate Household Income** | | | | | |
| < $50,000 | 142 (40%) | 68 (40%) | 26 (39%) | 13 (45%) | 3 (30%) | **252 (40%)** |
| $51,000 - $100,000 | 116 (33%) | 65 (38%) | 24 (36%) | 12 (41%) | 5 (50%) | **222 (35%)** |
| $101,000 - $200,000 | 70 (20%) | 26 (15%) | 12 (18%) | 2 (6.9%) | 1 (10%) | **111 (18%)** |
| > $200,000 | 13 (3.7%) | 4 (2.6%) | 0 (0%) | 0 (0%) | 0 (0%) | **17 (2.7%)** |
| Prefer not to answer | 11 (3.1%) | 9 (5.2%) | 5 (7.5%) | 2 (6.9%) | 1 (10%) | **28 (4.4%)** |
| **Total** | **352** | **172** | **67** | **29** | **10** | **630** |
|  |  |  |  |  |  | **p=0.950**^§^ |
|  | **Highest Level of Education** | | | | | |
| High School | 109 (31%) | 45 (26%) | 16 (24%) | 12 (41%) | 5 (50%) | **187 (30%)** |
| Community College | 102 (29%) | 53 (31%) | 24 (36%) | 10 (34%) | 3 (30%) | **192 (30%)** |
| University degree | 141 (40%) | 74 (43%) | 27 (40%) | 7 (24%) | 2 (20%) | **251 (40%)** |
| **Total** | **352** | **172** | **67** | **29** | **10** | **630** |
|  |  |  |  |  |  | **p=0.370** |
|  | **Participant Group** | | | | | |
| Canada | 175 (50%) | 93 (54%) | 25 (37%) | 17 (59%) | 5 (50%) | **315 (50%)** |
| US | 177 (50%) | 79 (46%) | 42 (63%) | 12 (41%) | 5 (50%) | **315 (50%)** |
| **Total** | **352** | **172** | **67** | **29** | **10** | **630** |
|  |  |  |  |  |  | **p=0.176** |
| * *P*-values significant at p<0.01  ^†^ Percentages in the columns may not add up to 100 due to rounding | | | | | | |
