# Supplementary material for: Knowledge, attitudes and influencers of cat owners in North America around antimicrobials and antimicrobial stewardship
Source: J Feline Med Surg. 2022 Apr 26;24(6):e90–7. doi: 10.1177/1098612X221090456 (PMC9161435; doi:10.1177/1098612X221090456)
Supplement: Table 2 [file sj-docx-2-jfm-10.1177_1098612X221090456.docx]

Supplementary table 2. Participant response to the question ‘Do you think antibiotic use in pets poses a risk to people?’ from a survey to assess knowledge, attitudes and influencers of cat owners in North America around antimicrobials and antimicrobial stewardship summarised by demographic group. The *p* values demonstrate differences in distributions of response across the demographic groups, as calculated by Pearson’s χ^2^ tests.

|  | **Yes** | **Might or might not** | **No** | **Total** |
| --- | --- | --- | --- | --- |
|  | **Gender** | | | |
| Female | 69 (51%) | 148 (68%) | 189 (68%) | **406 (65%)** |
| Male | 65 (49%) | 70 (32%) | 87 (32%) | **222 (35%)** |
| **Total** | **134** | **218** | **276** | **628** |
|  |  |  |  | **p<0.002** |
|  | **Age** | | | |
| 18-25 years old | 11 (8.2%) | 20 (9.1%) | 18 (6.5%) | **49 (7.8%)** |
| 26-35 years old | 36 (27%) | 45 (20%) | 54 (16%) | **126 (20%)** |
| 36-50 years old | 50 (37%) | 64 (29%) | 79 (29%) | **193 (31%)** |
| 51-65 years old | 27 (20%) | 62 (28%) | 95 (34%) | **184 (29%)** |
| > 65 years old | 10 (7.5%) | 29 (13%) | 39 (14%) | **78 (12%)** |
| **Total** | **134** | **220** | **276** | **630** |
|  |  |  |  | **p=0.018** |
|  | **Approximate Household Income** | | | |
| < $50,000 | 40 (30%) | 90 (40%) | 122 (44%) | **252 (40%)** |
| $51,000 - $100,000 | 47 (35%) | 74 (34%) | 101 (37%) | **222 (35%)** |
| $101,000 - $200,000 | 35 (26%) | 38 (17%) | 38 (14%) | **111 (18%)** |
| > $200,000 | 9 (6.7%) | 4 (1.8%) | 4 (1.4%) | **17 (2.7%)** |
| Prefer not to answer | 3 (2.2%) | 14 (6.4%) | 11 (4.0%) | **28 (4.4%)** |
| **Total** | **134** | **220** | **276** | **630** |
|  |  |  |  | **p<0.001** |
|  | **Highest Level of Education** | | | |
| High School | 26 (19%) | 69 (31%) | 92 (33%) | **187 (30%)** |
| Community College | 31 (23%) | 63 (29%) | 98 (36%) | **192 (30%)** |
| University degree | 77 (57%) | 88 (40%) | 86 (31%) | **251 (40%)** |
| **Total** | **134** | **220** | **276** | **630** |
|  |  |  |  | **p<0.001** |
|  | **Participant Group** | | | |
| Canada | 59 (44%) | 103 (47%) | 153 (55%) | **315 (50%)** |
| US | 75 (56%) | 117 (53%) | 123 (45%) | **315 (50%)** |
| **Total** | **134** | **220** | **276** | **630** |
|  |  |  |  | **p=0.048** |
| * *P*-values significant at p<0.01  ^†^ Percentages in the columns may not add up to 100 due to rounding | | | | |
